# Bodily Illusions and Motor Imagery in Fibromyalgia

**DOI:** 10.3389/fnhum.2021.798912

**Published:** 2022-01-20

**Authors:** Michele Scandola, Giorgia Pietroni, Gabriella Landuzzi, Enrico Polati, Vittorio Schweiger, Valentina Moro

**Affiliations:** ^1^NPSY-Lab.VR, Department of Human Sciences, University of Verona, Verona, Italy; ^2^Department of Human Sciences, University of Verona, Verona, Italy; ^3^Department of Surgery, Dentistry, Paediatrics and Gynaecology, University of Verona, Verona, Italy

**Keywords:** body representations, chronic pain, chronic pain and fibromyalgia, disturbed sense of ownership, action representation, bodily self, anxiety and depression

## Abstract

Fibromyalgia (FM) is characterised by chronic, continuous, widespread pain, often associated with a sense of fatigue, non-restorative sleep and physical exhaustion. Due to the nature of this condition and the absence of other neurological issues potentially able to induce disorders in body representations per se, it represents a perfect model since it provides an opportunity to study the relationship between pain and the bodily self. Corporeal illusions were investigated in 60 participants with or without a diagnosis of FM by means of an *ad hoc* devised interview. In addition, motor imagery was investigated and illusions relating to body part movements and changes in body size, feelings of alienness, and sensations of body parts not belonging to one’s own body (disownership and somatoparaphrenic-like sensations) were found. Crucially, these symptoms do not correlate with any of the clinical measures of pain or functional deficits. The results showed that motor imagery was also impaired, and the severity of the deficits found correlated with the functional impairment of the participant. This indicates that disorders in body representations and motor imagery are part of the clinical expression of FM. However, while motor imagery seems to be linked to reduced autonomy and functional deficits, bodily illusions are independent and potentially represent a concurrent symptom.

## Introduction

Although perceived by individuals as unique and consistent, the sense of self is a complex construct which is built over the course of a person’s life on the basis of affective and social experiences, cognition (e.g., memories) and sensory-motor activity. A component of the sense of self is the bodily self, that is, the contemporaneous experiences of the presence of a body, having that body and also being the same body. In other words, we sense that the body we inhabit is our own body and that we are that body. This experience is taken for granted in daily life and is not something which we pay attention to unless something particular or unexpected happens (e.g., when your arm falls asleep due to the compression of peripheral nerves).

In spite of this apparent stability, the sense of body is extremely plastic and fragile, as shown in several studies on neurological patients with body representation disorders as a consequence of brain damage (Pacella et al., 2019; Fornia et al., 2020; Moro et al., 2021b). Furthermore, experiments with healthy participants indicate that even in the absence of a brain lesion, information coming from the body may be delusive and induce corporeal illusions (Botvinick and Cohen, 1998; Pavani et al., 2000; Maravita et al., 2003; Tsakiris and Haggard, 2005; Pavani and Zampini, 2007; Tsakiris et al., 2010; Tieri et al., 2015a,b; Golaszewski et al., 2021). These experiments used multisensory integration in order to mislead the brain in its attempt to interpret sensory information which is processed as coming from one’s own body although in fact they come from non-bodily sources. Two examples of this type of experiment are the rubber hand illusion (Botvinick and Cohen, 1998) and the enfacement illusion (Porciello et al., 2018) in which people have reported the sensation that a rubber hand or another person’s face have become their own hand or face when they received a tactile stimulation that was synchronous (and spatially congruent) with another stimulus which appeared to be administered to the rubber hand or the other person’s face. In particular, temporal synchrony is crucial in both rubber hand (Costantini et al., 2016; Motyka and Litwin, 2019) and enfacement illusions (Tajadura-Jiménez and Tsakiris, 2014; Apps et al., 2015).

This supports the idea that sensory information plays a crucial role in bodily self, as shown by studies on deafferented people, in particular individuals suffering from spinal cord injury (SCI; Scandola et al., 2014, 2017a,2019b; Moro et al., 2021a) or locked-in-syndrome (Nizzi et al., 2012), and recently confirmed by the effects of interoceptive modulations on the sense of body (Salvato et al., 2017; Jenkinson et al., 2020; Monti et al., 2020; Todd et al., 2021).Thus, representations underlying bodily self are the result of a continuous integration of multiple sources of information. This integration process provides immediate feedback on the current state of the body and is also integrated with higher-order cognitive functions (e.g., spatial perception and memory) in order to obtain a detailed map of the body and its relationship with the environment in terms of action planning and motor imagery. Although authors on the subject agree on this point (namely, that body representations are the complex result of multiple components, see Berlucchi and Aglioti, 2010), the contribution of the individual components still needs to be disambiguated. For example, the potential role of pain involved in the sense of body remains for the most part unknown (see Haggard et al., 2013 for a review).

From a naïve perspective, one might be led to think that pain has a detrimental effect on body representations. Indeed, painful conditions alter the threshold of sensitivity (Le Bars et al., 1979) and reduce the ability to localise other sensory stimuli (Haggard et al., 2013; Osumi et al., 2015). Nevertheless, if chronic pain has been found to be commonly associated with a reduced tactile sensitivity of the affected part (Moriwaki and Yuge, 1999; Pleger et al., 2006), acute pain seems to facilitate the perception of touch (Ploner et al., 2004). In addition, in chronic syndromes (i.e., complex regional pain syndrome), pain impacts on the mental imagery of movement, but only when the actions relate to the painful body parts (Schwoebel et al., 2001; Schwoebel and Coslett, 2005; Martínez et al., 2018). The results of this research indicate a link between pain, body representations and motor imagery.

Furthermore, the results of experimentation with deafferented people suffering from spinal cord injury support the presence of such a link and indicate the possibility of differentiated effects of pain on body representations and motor imagery (Gustin et al., 2008; Soler et al., 2010, 2021; Kumru et al., 2013; Scandola et al., 2017a,b). In particular, musculoskeletal, neuropathic, and visceral pain all seem to contribute to misperceptions of body parts (e.g., the feeling of having some body parts in a position which is different to the actual position), while the presence of neuropathic pain might have a “protective” effect on body representations, reducing the feeling of illusory movements of body parts (i.e., sensations of motion that are not voluntarily controlled and muscular fatigue after illusiory movements). However, it is worth noting that in spinal cord injured people, pain is not the main, distinctive symptom, since the massive deafferentation and deefferentation which these people suffer from may cause body representation disorders *per se*.

Fibromyalgia (FM) is a clinical condition that provides an opportunity to study the specific impact of pain on body representations. It is a chronic musculoskeletal condition characterised by widespread pain and evoked pain at tender points (Wolfe et al., 2016). Other typical symptoms are a sense of fatigue, sleep disorders and non-restorative sleep. Physical exhaustion and cognitive difficulties, in particular involving the individual’s memory, have also been reported. In addition, most patients report a wide range of additional somatic and psychological symptoms, such as rheumatic disorders, irritable bowel syndrome, as well as anxiety and depression (Häuser et al., 2015). As a consequence, FM negatively impacts psychological wellbeing, social relationships and work life (Chinn et al., 2016; Andrade et al., 2020). In industrially developed countries, up to 5.8% of the population present with a diagnosis of FM (Branco et al., 2010; Ablin et al., 2012; Wolfe et al., 2013), with a much higher prevalence in the case of women (8–10:1, female to male ratio). The aetiology is to date unknown but appears to be centred on genetic, environmental and personality contributors (Furness et al., 2018).

There are a number of reasons why FM represents a good model for a study of the effects of pain on body representations. Firstly, it is a chronic condition with symptoms that last over time with a certain degree of stability. Patients typically report continuous pain that they remember accompanying them from childhood or youth (although the diagnosis is more frequently made when the patient is between 40 and 60 years of age; Häuser and Fitzcharles, 2018) and it becomes a constant presence in their life. In terms of the connection discussed above, it appears that this condition extends its effects onto the bodily self, as indicated by previous results of research into chronic pain (Moseley, 2004; Moseley et al., 2012; Akkaya et al., 2013; Tsay et al., 2015). Another crucial feature is that FM is not associated with neurological deficits or specific lesions in the central nervous system that might cause direct modifications in cognitive representations of the body or alterations to sensory-motor systems. This makes it possible to investigate directly whether potential disorders in body and motor representations are a consequence of pain (without other causes) or if they may represent specific, independent aspects of the syndrome. Finally, FM tends to affect young people (in particular women) who are attentive to signals coming from their body and are able to describe these accurately giving detailed reports.

In this study, we capitalised on previous results coming from a specific battery of tests which investigated various different components of body representation (Scandola et al., 2017a). These were administered to a sample of women, with or without a diagnosis of FM, in order to determine the potential effects of chronic pain on the bodily self. In addition, we analysed the potential relationships between clinical symptoms, body representations and the ability to represent actions (i.e., motor imagery). The effects of personality traits and clinical variables such as the severity of the symptoms, the various different typologies of pain (musculoskeletal, neuropathic, and visceral pain) and the time interval since the onset of symptoms were also considered. The results are potentially useful not only in terms of the comprehension of the various facets of the bodily self, but also with regard to the clinical impact on diagnosis and interventions for FM.

## Materials and Methods

### Participants

Sixty women participated in the study over a period of one year, 30 with a diagnosis of FM, who were recruited at the Analgesic Therapy Unit, Borgo Roma Hospital, Verona during their periodic medical visits, and 30 healthy controls (C) who were recruited through the friends and contacts of the experimenters. No men were recruited because of the low prevalence of FM in males. The two groups were matched for age [FM: *M* (SD) = 48.45 (10.92), C: *M* (SD) = 49.9 (10.22), Mann–Whitney *U* = 397.5, *p* = 0.441, *r* = 0.1) but not standard of education [FM: *M* (SD) = 12.3 (3.505), C: *M* (SD) = 14.77 (2.582), *U* = 269.5, *p* = 0.005, *r* = 0.36]. The clinical diagnosis of FM was confirmed or excluded by means of combining the individuals’ scores on the Widespread Pain Index (WPI) and the Symptom Severity Scale (SSS; Wolfe et al., 2016, 2018). The WPI is a self-reported pain index resulting from the summary count of the number of painful regions out of the 19 regions considered in the Regional Pain Scale (Wolfe, 2003) (score range = 0–19). The SSS is the sum (range 0–12) of the severity scores of three symptoms (i.e., fatigue, waking unrefreshed, and cognitive symptoms, score range for each item = 0–3), along with the sum of the number of other symptoms that might have co-occurred during the previous 6 months (i.e. headaches, pain or cramps in the lower abdomen, and depression, score 0–3) (Wolfe et al., 2018). Combinations of WPI ≥ 7 and SSS ≥ 5 or WPI ≥ 4 and SSS ≥ 9 were considered as two valid cut-offs for a diagnosis of FM. This meant in fact that, in the FM group, pain was present in at least 4 or 5 regions (with jaw, chest, and abdominal pain not included in the list) and symptoms had been present for at least 3 months. It is to be noted that, following these diagnostical criteria (Wolfe et al., 2018), the diagnosis of FM is considered to be valid irrespective of other diagnoses and does not exclude the presence of other serious clinical illnesses (Wolfe et al., 2016, 2018).

The participants in the control group were recruited through experimenters’ personal contacts and each FM patient was matched with a control of the same age (±5 years). In this group, the presence of FM was excluded by means of the WPI and SSS scores.

### Preliminary Clinical Assessment

To investigate in depth the nature of the pain that had been reported by the FM participants, and in particular to ascertain whether they reported musculoskeletal, visceral, or neuropathic pain differently from each other, we analysed the participants’ responses (both FM and C) to a questionnaire with a scale previously devised in our laboratory (Scandola et al., 2017b). The validation of this scale (higher scores, more severe symptoms) is currently in progress, but preliminary results confirm a high correlation with both the Brief Pain inventory (BFI; Caraceni et al., 1996) and the Douleur Neuropathique 4 question scale (Bouhassira et al., 2005; Scandola et al., 2017b). For the purposes of the analyses carried out in this case, we have only considered the scores indicated by the participants as reflecting the degree of each typology of pain in the worst moment of pain that they could remember. Details of the characteristics of the various typologies of pain and the frequency of each of these features in the two groups are shown in the Supplementary Material (Supplementary Table 1).

A check for the presence of potential disorders in mood was also carried out by means of the Hospital Anxiety and Depression Scale (Bjelland et al., 2002), a self-administered scale that gives scores separately for anxiety and depression. 14 multiple-choice questions are asked that investigate the frequency (1 = never, 5 = always) with which a specific mood occurs (e.g., “I feel agitated and tense” “I feel in a good mood”).

Finally, in order to consider the impact of pain on daily life activities, the Italian version of the Fibromyalgia Impact Questionnaire (FIQ; Sarzi-Puttini et al., 2003) was administered. This questionnaire is divided into three parts which focus on the condition of the patient as perceived subjectively during the week before the assessment. The questions refer to: (i) the patient’s ability to perform daily tasks involving the large muscles (e.g., shopping, housework, gardening); (ii) the number of days in the past week that they felt good and the number of days that they missed work because of pain; and (iii) the intensity of symptoms such as pain, fatigue, morning tiredness, stiffness, anxiety, depression, and the ability to do one’s job. The scores for the three parts are then normalised to give a total score ranging from 0 to 100, with higher scores indicating worse conditions (for details, see Sarzi-Puttini et al., 2003).

The scores of the two groups on these scales and other clinical data are reported in Table 1 and confirm the differences between the two groups in all of the measures relating to FM (WPI, SSS, and FIQ) and in all of the three typologies of pain (musculoskeletal, visceral, and neuropathic pain).

**TABLE 1:** Results of the clinical assessment of the two groups.

	FM	C	Frequentist test	*p*	Effect size
WPI	13.47 (3.87)	3.233 (2.34)	*U* = 893	<0.001***	*r* = 0.85
SSS	9.53 (1.48)	4.2 (2.5)	*U* = 883	<0.001***	*r* = 0.83
FIQ	65.34 (21)	16.616 (9.47)	*U* = 877.5	<0.001***	*r* = 0.82
Interval (y)	12 (range 1–39)	–	–		
Motor activity (%)	43.33	60	χ^2^_(1)_ = 1.07	0.30	*V* = 0.17
Job (%)	83.33	86.67	χ^2^_(1)_ = 0.00	1	*V* = 0.05
musculoskeletal pain	8.77 (1.5)	6 (3.21)	*U* = 706	<0.001***	*r* = 0.5
visceral pain	7.2 (3.17)	1.63 (2.91)	*U* = 778	<0.001***	*r* = 0.66
neuropathic pain	5.83 (4.32)	1.83 (3.37)	*U* = 671.5	<0.001***	*r* = 0.47
HADS Anxiety	9.33 (3.6)	9.83 (3.35)	*U* = 375	0.27	*r* = 0.14
HADS Depression	5.47 (3.85)	4.1 (2.41)	*U* = 526	0.26	*r* = 0.15

FM, mean and standard deviation for the FM group. C, mean and standard deviation for the C group; Frequentist test, the score at the frequentist statistical test used; U, Mann–Whitney non-parametric t-test. Interval, time interval from the appearance of symptoms to the assessment; Y, years; %, the percentage of participants who have a job and who regularly practice motor activity (at least three times per week) are reported; r, rank-biserial effect size (small effect = r < 0.3; moderate effect = r < 0.5; large effect = r ≥ 0.5; Tomczak and Tomczak, 2014); V, Cramer’s V effect size (moderate = 0.2 < V ≤ 0.6, large V > 0.6; Rea and Parker, 2014). ***p < 0.001.

Other information, such as the patient’s work situation (FM = 25 out of 30, C = 26 out of 30; χ^2^_(1)_ = 0; *p* = 1; Cramer’s *V* = 0.05) and whether they regularly do motor activity (at least three times per week, FM = 13 out of 30, C = 18 out of 30; χ^2^_(1)_ = 1.07; *p* = 0.30; Cramer’s *V* = 0.17) were recorded, with no differences in frequency between the groups. Finally, it is noteworthy that the groups did not show any differences relating to anxiety and depression. The presence of correlations between these clinical variables and any symptoms of corporeal illusions or motor imagery deficits was also assessed (see below).

### The Evaluation of Body Representation Disorders

The Body Feelings and Illusions questionnaire (BoFI; Scandola et al., 2017a) was administered to the two groups of participants. The questionnaire is comprised of 22 questions each of which investigate the presence or absence (score 1–0) of any symptoms related to Body form and integrity (Q1.1–1.13) or any feelings relating to Body part positions or illusory movements (Q2.1–2.8). When a symptom was reported, further questions were asked about the situations in which the feeling occurred and its qualitative characteristics. Based on a Principal Component Analysis carried out in a previous validation of the questionnaire (Scandola et al., 2017a), the majority of the questions fall into specific components (see Table 2) such as: feelings of *Body loss* (i.e., sensations of body parts disappearing or missing); *Illusory motion* (i.e., sensations of motion that are not voluntarily controlled with muscular fatigue after illusionary movements); *Body part misperception* (i.e., the feeling of having some body parts in a position which is different to the actual position); *Aversive feelings* (i.e., negative feelings toward a given body part; in the original study this was called Misoplegia to describe symptoms reported by spinal cord injured people who are paralysed in the lower lesioned body parts); *Disownership-like feelings* (i.e., the feeling that body parts do not belong to the person) and *Somatoparaphrenia-like sensations* (i.e., the feeling that body parts are “alien” or detached from the body).

**TABLE 2:** The body feelings and illusions questionnaire.

Body Feelings and Illusions		Which and where?
1	Have you ever felt strange sensations in your body?	
	Body form and integrity	
1.1.	Does it ever feel like any body parts do not belong to you? (DSO)	
1.2.	Does it ever feel like your arms are not attached to your shoulders? (DSO)	
1.3.	Does it ever feel like your legs are not attached to your hips? (SP)	
1.4.	Do you ever feel like your legs/arms are elsewhere in the room/in space?	
1.5.	Do you ever feel that a part of your body (e.g., your arms or legs) are missing? (BL)	
1.6	Do you ever feel that a part of your body (e.g., your arms or legs) have disappeared? (BL)	
1.7.	Do you ever feel that your legs have become longer?	
1.8.	Do you ever feel that your arms have become longer?	
1.9	Do you ever feel any body parts swelling (IM)	
1.10	Do you ever feel like any parts of your body have become smaller?	
1.11	Do you ever feel the desire not to have a particular body part? (BL/MP)	
1.12	Does it ever feel like some body parts are alien or foreign? (SP)	
1.13	Do you ever feel hate for any body parts? (MP)	
	Body and body part positions and illusory movements	
2.1	Does it ever feel like any parts of your body (e.g., arms or legs) are in a different position with respect to your real posture? (BPM)	
2.2.	Does it ever feel like you are in a different position with respect to your real posture? (BL)	
2.3.	Des it ever feel like your knees and hips are bent when instead they are totally extended? (BPM)	
2.4.	Does it ever feel like your toes are in a strange position, for example curved inwards? (SP)	
2.5.	Does it ever feel like any body parts move involuntarily? (IM)	
2.6.	Do you ever have the feeling that your muscles are moving with subsequent tiredness? (IM)	
2.7	Does it ever feel like each digit was twisted so that each toe or finger points in a different direction?	
2.8.	Does it ever feel like your fingers or toes are clenched or overlapping one other? (BMP)	

*Following Scandola et al.’s (2017a) validation of the scale, each question falls into one of six factors indicated in brackets. DSO, disownership-like sensations; SP, somatoparaphrenia-like sensations; BL, body loss; IM, illlusory movements; MP, aversive feelings (Misoplegia, in the original version); BPM, body part misperceptions; six questions (in light gray, n.1; 1.4; 1.7; 1.8; 1.10; 2.7) do not fall into specific components as the PCA indicates (Scandola et al., 2017a).*

The scores of each component were computed as the sum of the responses to the questions with positive loadings to the component, minus the responses to the questions with negative loadings. Some questions do not fall into any of the components but were, however, administered, even though they were not considered in the statistical analyses (Table 2).

### The Assessment of Motor Imagery

Two subscales of the Visual Motor Imagery Questionnaire-2 (Isaac et al., 1986; Roberts et al., 2008) were used to assess motor imagery abilities from first and third person perspectives. More specifically, the subscale of External Visual Imagery (VMIQ-EVI) necessitates imaging oneself performing actions from a third person perspective (“as if you were watching yourself from an external position”). In contrast, the first person perspective imagery was assessed by means of the Kinesthetic Imagery scale (VMIQ-KIN) with the participants being asked to “imagine feeling themselves performing the movement.” Thus, the two subscales involve different cognitive processes, specifically visual imagery in the case of the former and simulation of bodily sensations for the latter (Ionta et al., 2010; Scandola et al., 2017b; Moro et al., 2021a). The vividness of each action that they imagined (12 identical actions in the two subscales) was rated by the participant on a 5-point Likert scale (with 1 = perfectly vivid imagined action and 5 = not imagined at all). The sums of the scores were considered as the final scores for the two subscales (maximum score = 60) (Table 3).

**TABLE 3:** The Visual Motor Imagery Questionnaire (Roberts et al., 2008).

	Kineasthetic (“how well can you imagine feeling yourself doing this movement?”)					Visual (“how well can you see yourself doing this movement from a third-person perspective?”)				
	1	2	3	4	5	1	2	3	4	5
Walking										
Running										
Kicking a stone										
Bending to pick up a coin										
Running upstairs										
Jumping sideways										
Throwing a stone into water										
Kicking a ball in the air										
Running downhill										
Riding a bike										
Swinging on a rope										
Jumping off a high wall										

*The 12 items and the two conditions of assessment are shown. 1 = Perfectly clear and vivid as a normal vision; 2 = clear and reasonably vivid; 3 = Moderately clear and vivid; 4 = Vague and dim; 5 = No image at all, you only know that you are thinking of the skill.*

### Procedure

The participants were recruited during their follow-up visits which included the administration of WPI, SSS, and FIQ. They were informed of the aims of the study and were requested to sign the informed consent form. They were then interviewed in a quiet room in the Anthalgic Therapy Unit (Department of Surgery, Dentistry, Paediatrics and Gynaecology) by two examiners (GP and VM or MS), with one asking the questions and the other transcribing the answers. First, a clinical assessment was carried out using the Verona Pain Scale, HADS and a number of questions regarding motor activity and the participant’s employment situation. These tasks were administered in a counterbalanced order. Subsequently, the BoFI and VMIQ (VMIQ-EVI and VMIQ-KIN) were executed in a counterbalanced order. The whole session lasted from 30 to 45 min, depending on the dialogue that was created between the participant and the examiners and the number of symptoms and details to be recorded.

### Analysis of Data

The experimental variables were not parametrically distributed (all Shapiro–Wilk tests *p* < 0.05), thus, in order to compare the FM and C groups, non-parametric tests were used. When a variable was continuous, the Mann–Whitney test for independent samples was used, using the *r* rank-biserial correlation as the measure of the effect size. This ranged between 0 and 1 (small effect = *r* < 0.3; moderate effect = *r* < 0.5; large effect = *r* ≥ 0.5; Tomczak and Tomczak, 2014). When a variable was scored as a frequency, a χ^2^ test was used with Cramer’s *V* as effect size (moderate = 0.2 < *V* ≤ 0.6, large *V* > 0.6; Rea and Parker, 2014). Furthermore, in order to assess any potential association between bodily illusions and clinical variables, a series of correlations were executed separately for the two groups. All of the statistical analyses were executed by means of the R statistical framework (R Core Team, 2020).

## Results

### Body Representation Disorders

The results of the comparison between the FM and C groups are reported in Table 4. They indicate that in the case of the FM group, feelings of Illusory Motion, Disownership-like sensations and Somatoparaphrenia-like sensations, as well as Aversive feelings are more frequent than in the C group. No differences were recorded for feelings of Body Loss and Body part Misperception. The FM group did not perform as well as the C group in terms of motor imagery, both in the VMIQ-KIN and VMIQ-EVI subscales.

**TABLE 4:** The comparison of the two groups in the BoFI components and VMIQ questionnaires.

	FM	C	Test	*p*	*r*	Total
Body loss	0.067 (0.74)	–0.167 (0.461)	*U* = 531.5	0.167	0.18	–0.05 (0.622)
**Illusory motion**	1.567 (0.971)	0.667 (0.606)	*U* = 682	**<0.001**	0.48	1.117 (0.922)
Body part Misperception	0.267 (0.785)	0.133 (0.434)	*U* = 490	0.483	0.09	0.2 (0.632)
**Aversive feelings**	1.233 (0.774)	0.7 (0.837)	*U* = 607	**0.014**	0.32	0.967 (0.843)
**Disownership**	1.033 (0.809)	0.133 (0.434)	*U* = 724.5	**<0.001**	0.6	0.583 (0.787)
**Somatoparaphrenia**	1 (0.91)	0.167 (0.461)	*U* = 686	**<0.001**	0.52	0.583 (0.829)
**VMIQ—EVI**	40.333 (12.949)	26.633 (9.775)	*U* = 707	**<0.001**	0.49	33.483 (13.308)
**VMIQ—KIN**	41.8 (12.093)	28.933 (12.706)	*U* = 701.5	**<0.001**	0.48	35.367 (13.904)

*FM = mean and standard deviation of the FM group; C = mean and standard deviation of the C group; Test t = the value of the Mann-Whitney statistical test; p = the p-value, statistically significant differences are in bold; r = the rank-biserial effect size; Total = the overall mean and standard deviation.*

A statistical analysis of the responses (details of the frequencies of the responses to individual items are shown in Supplementary Table 2) indicates that, among the questions falling into the category of *Illusory motion* components, the feeling of illusory muscular work, with a consequent sensation of fatigue (Q2.6) is present in 26.67% of the FM participants, while in the C group, this was only reported by one participant (χ^2^_(1)_ = 4.71, *p* = 0.03, Cramer’s *V* = 0.33). Some FM patients reported this sensation involving the whole body and other that it was localised to their limbs and hands. In the same component category, the feeling that some body parts involuntarily move (Q2.5, legs and arms in particular) was present in 43.33% of the FM participants, while in the C group this was reported by 20% of the participants, who, however, reported feelings of muscular fasciculation rather than limb movements. Even if the results relating to this sensation are not statistically different between the two groups (χ^2^_(1)_ = 2.77, *p* = 0.10, Cramer’s *V* = 0.25), there is a qualitative difference. Indeed, if we remove the feeling of muscular fasciculation from both groups and consider only actual sensations of body part movement, the difference becomes statistically significant (χ^2^_(1)_ = 7.55, *p* = 0.006, Cramer’s *V* = 0.39).

Finally, a sensation of body part swelling (Q1.9) was more frequent in the FM group (87%) than in the C group (43%) (χ^2^_(1)_ = 10.55, *p* < 0.001, Cramer’s *V* = 0.45). This sensation specifically involves the hands and feet and sometimes an entire limb. In five patients, sensations of swelling were reported in the abdomen and four participants reported the sensation involving their face. Sensations of swelling were also present in the C group with a number of them claiming that they had experienced this, but not so frequently.

With regard to *Disownership-like sensations*, the FM participants reported both sensations that certain body parts did not belong to their body (Q1.1, 56.67%) and that their arms were not attached (Q1.2, 46.67%), feelings which were rare or absent in the C group (10 and 3.33%, respectively). In both cases, the differences were statistically significant (χ^2^_(1)_ = 12.67, *p* < 0.001, Cramer’s V = 0.5; χ^2^_(1)_ = 12.8, *p* < 0.001, Cramer’s *V* = 0.5, respectively). These sensations commonly involve the limbs, but one of the FM participants reported this sensation in the head, and two in their whole body. In particular, these seem to occur during stressful or anxious moments, but also when they are half-asleep, tired or at the moment when the pain is exacerbated.

The feeling of one’s legs not being attached (Q1.3, in the *Somatoparaphrenia-like sensations* component category) was frequent in the FM group (43.33%) but not in the C group (10%; χ^2^_(1)_ = 12.67, *p* < 0.001, Cramer’s *V* = 0.5). Specific sensations of alienness of body parts (Q1.12) were present in around a quarter of the FM participants (23%), but were not reported at all by the C participants (0%; χ^2^_(1)_ = 5.82, *p* = 0.016, Cramer’s *V* = 0.36). In this component category, there were also reports of sensations of the toes being in strange positions (Q2.4), as claimed by one third of the FM participants (33%) but only in a few cases in the C group (6.67%; χ^2^_(1)_ = 5.1, *p* = 0.024, Cramer’s *V* = 0.33).

Other frequent symptoms in the FM group referred to a desire not to have a particular body part (Q1.11; FM = 63.33%; C = 40%; χ^2^_(1)_ = 5.42, *p* = 0.001, Cramer’s *V* = 0.32) and a feeling of hate toward certain body parts Q1.13; FN = 60%; C = 30%; χ^2^_(1)_ = 4.31, *p* = 0.038, Cramer’s *V* = 0.3). These sensations are mostly felt toward painful body parts and, while in the C group they were limited in number and extremely specific, but the most relevant characteristic was that these painful sensations were constant or almost constant. (the pain sensations felt from people of the C group reporting hate toward the bodily parts were: toothache (three people), pain in the stomach, intestine and the pelvic floor (one person), pain in a buttock and a calf (one person), pain at shoulders and head (one person), pain in the right heel and hip (one person), pain in the left eye (one person); one person also associated pain with the feeling of restless legs during night), in the FM group they involved large areas of the body and sometimes the whole body. It is to be noted that, in the PCA (Scandola et al., 2017a), Q1.11 (regarding the desire not to have a particular body part) loads negatively in the Body Loss component and positively in the Aversive feeling component (see Table 2).

Within the BoFi-FM, the various components are partially correlated in the FM group, for which Illusory movements correlate with Body Loss (*r* = 0.43, *p* < 0.05), Disownership-like sensations (0.46, *p* < 0.05) and Somatoparaphrenia-like sensations (0.62, *p* < 0.001). These latter two components also correlate with each other (0.66, *p* < 0.001) and with Aversive feelings toward body parts (0.49, *p* < 0.01). In the C group, only Somatoparaphrenic and Disownership-like sensations were correlated (0.57, *p* < 0.001). The complete results are reported in Supplementary Tables 3A,B.

### Effects of the Clinical Variables on Body Representation Disorders

In contrast to our expectations, we did not find any specific correlations between clinical variables (WPI, SSS, pain severity, interval from symptoms onset and diagnosis, and functional deficits at the FIQ) and corporeal illusions in the FM group, but there was an inverse correlation between age and Body loss: –0.5, *p* < 0.01. In the C group, the severity of pain symptoms (as measured by the SSS) correlated with some components such as Body loss (–0.39, *p* < 0.05), Illusory movement (0.39, *p* < 0.05), and Aversive feelings (0.49, *p* < 0.01). In this latter group, a correlation between standard of education and somatoparaphrenic-like sensations was also found (0.57, *p* < 0.001).

### Motor Imagery

Patients with FM show disorders in motor imagery in both the visual and kinaesthetic conditions as compared to the matched controls. In the FM group, the two subscales correlated with each other and also with the severity of the symptoms as measured by the FIQ (VMIQ-EVI: 0.38, *p* < 0.05; VMIQ-KIN: 0.42, *p* < 0.05). A negative correlation was found between the VMIQ-EVI and the duration of the illness (i.e., the interval of time between the first symptom and the assessment, –0.39, *p* < 0.01), indicating that this capacity deteriorates over time. In the C group, the VMIQ KIN correlated with all the measures of pain and functional deficits (FIQ: 0.44, *p* < 0.05, SSS: 0.52, *p* < 0.52), while the VMIQ EVI correlated with WPI (0.36, *p* < 0.05) and SSS (0.67, *p* < 0.001).

### Correlations Between Clinical Variables

In the FM group, anxiety and depression did not correlate with other clinical variables or with bodily misperceptions and motor imagery, while in the C group anxiety was associated with WPI (0.37, *p* < 0.05).

## Discussion

In the present study, the BoFI questionnaire was used to investigate the presence of spontaneous sensations and misperceptions relating to the body. The main result regards the evidence that was found that bodily self is compromised in FM, although with symptoms that are more specific than in other clinical conditions such as strokes (Romano and Maravita, 2019) or deafferentation and deefferentation due to spinal cord injuries (Scandola et al., 2017a). Indeed, while in these conditions corporeal illusions involve the feeling of the presence of the body itself (Jenkinson et al., 2018), the FM patients did not report sensations of Body loss more frequently than the controls (i.e., sensations of body parts disappearing or missing) or misperception of body parts (i.e., the feeling of having some body parts in a position which is different to the actual position). Corporeal illusions seem to be specifically associated with a sense of strangeness and alienness of the body, in other words, giving a sense of being in their body and having their body. In fact, they reported the impression of illusory motion and disownership or somatoparaphrenic-like sensations, that is, the feelings that body parts do not belong to them or are “alien”. The presence of Aversive feelings (Misoplegia) is consistent with previous data indicating lower levels of acceptance regarding painful body parts (Martínez et al., 2018).

A second interesting, unexpected result is the lack of correlations between these bodily sensations and pain. This suggests the possibility that alterations in the bodily self represent a specific feature of the syndrome which is not secondary to other clinical aspects. Although Aversive feelings toward the body were reported, pain and body sensations were independent from mood, in particular from anxiety and depression in the FM group, unlike in the case of the controls for whom the extent of the pain they experienced (as measured by WPI) correlated with the degree of anxiety.

Finally, a reduced capacity with regard to motor imagery was found in the FM group, both when the task was executed from a first person perspective (with an internal simulation of the movement—VMIQ-KIN) and a third person perspective (with the participant imaging as seing themselves in a mirror while performing the action—VMIQ-EV). Motor imagery disorders correlated with functional deficits (FIQ) in both groups. Furthermore, an effect of mood was reported, since in the FM group both of the results of the motor imagery tasks correlated with depression.

### Disorders in Body Representations in Fibromyalgia

Body representation may be investigated by means of various methodologies, and in FM a number of different approaches have been used, ranging from interviews and illness narratives (Valenzuela-Moguillansky et al., 2013), to scales and questionnaires (Akkaya et al., 2013; Rost et al., 2017) and experimental procedures (Valenzuela-Moguillansky et al., 2017; Martínez et al., 2018, 2019). In the present study, a specific questionnaire was used to investigate the presence of spontaneous sensations and misperceptions relating to the body. This allowed us to distinguish various different aspects involving corporeal illusions. The hypothesis that these six components are independent of each other was supported by the results from the control group which indicate that they do not correlate with each other, with the exception of Disownership and Somatoparaphrenic-like sensations. In contrast, in the FM group, correlations between Illusory movements and feelings of Body loss, Disownership and Somatoparaphrenic-like sensations were found, and these latter correlated with Aversive responses (Misoplegia).

Phenomena related to sensations of illusory motion, with involuntary motion and consequent muscular fatigue, such as those recorded with the Illusory Motion subscale, previously been reported in patients suffering from neurological disorders (Jenkinson et al., 2015; Scandola et al., 2017a). In cases of FM, the most frequent symptom involves sensations of swelling in certain body parts, which is also associated with body size perception. This result confirms previous studies in these patients, showing difficulties in estimating their own body size, with enlarged body size perception and shrinkage of the surrounding space, in particular when pain is exacerbated (Valenzuela-Moguillansky et al., 2013). In an experimental study, a body-scaled action anticipation task was used, in which the participants were asked to estimate whether they would be able to pass through apertures of varying widths that simulated doors. The “passability ratio” (i.e., the aperture size for a 50% positive response rate divided by the participant’s shoulder width) was higher for the FM participants, indicating an overestimation of their body size. This correlated positively with pain and functionality, but, as in our results, not with pain intensity (Valenzuela-Moguillansky et al., 2017).

A novelty in the present study regards evidence of feelings of detachment, alienness, and a reduced sense of ownership of body parts, such as the feeling that arms or legs are detached from the shoulders or hips or that these do not belong to one’s own body (Body loss, Disownership, and Somatoparaphrenic-like sensations subscales). Sometimes these feelings are present when there is pain, but they often occur in relaxed moments or when patients are resting in bed. Thus, a direct explanation based on the presence of pain cannot be drawn from our data.

Sensory information processing may contribute to these sensations. Distorted localisation of tactile stimulation (administered to painful sites) has been found in FM patients (Martínez et al., 2019) who report anomalous sensations (e.g., tingling, pins and needles, a feeling of heaviness, and cramps) as if they were emanating from somewhere away from the site being stimulated, sometimes within the same dermatome but often remotely or in another limb. Interoception (i.e., the processing of information coming from inside one’s own body) has also been investigated, with some experimental data indicating a perception of reduced heartbeat in FM patients (Borg et al., 2018) and other reports showing no difference with healthy subjects (Rost et al., 2017; Valenzuela-Moguillansky et al., 2017). It is thus plausible that altered information coming from the body contributes to corporeal illusions as an indirect result of neuroplastic processes. Interoception might also have a role in the lack of connection between chronic pain and bodily illusions. Indeed, interoception is an important source for body representations (Craig, 2003), that is altered in chronic pain conditions (Di Lernia et al., 2016), in particular in chronic visceral pain (Bonaz et al., 2021).

However, experimental studies suggest that other cognitive elements contribute, and these are associated more with high order, body representations. For example, FM patients performed significantly worse with respect to controls in a task involving specifying the laterality of a hand (Martínez et al., 2019). In this task, the participants look at images of hands (or feet) which are rotated to different degrees (e.g., 0°–90°–180°–270°), and they are asked to decide if the image corresponds with the right or left body part. When the participant activates body representation, the response times increase with the increase in the degree of rotation in the image (i.e., 180°). This indicates that the task is executed by means of a bodily simulation of the position of the body part. When the task is executed with a visual, rather than corporeal, strategy, the response times do not change with the degree of rotation. This is considered to represent an index of disorders in body representation and was recorded in cases of FM, as well as in other clinical conditions (Parsons, 1994; Ionta and Blanke, 2009; Conson et al., 2013; Scandola et al., 2019a). In line with this, FM patients seem to be more sensitive to the effects of the rubber hand illusion (Botvinick and Cohen, 1998). When their own hand and a rubber hand are synchronously stroked, the patients’ response is accentuated with respect to the controls, both in terms of the proprioceptive drift and the sense of ownership and motor control over the rubber hand (Martínez et al., 2018). Taken together, these results suggest that a feature of the FM syndrome regards the fragility of one’s own body, characterised by instability regarding self-body representations and a greater sensitivity to illusions. Rather than being a consequence of pain, disorders relating to the bodily self might represent a concurrent symptom (Martínez et al., 2018).

Neuroimaging studies support this hypothesis. Although in fMRI brain activity during touch is similar in FM patients and controls, patients show differences when they are asked to rate pleasure and pain with deactivation and hyperactivation in the posterior insular contralateral to the stimulated arm, respectively (Boehme et al., 2020). Voxel-brain-morphometry analyses also reveal reduced gray matter density in the anterior insula and hippocampus (Boehme et al., 2020). Finally, alterations in the connectivity of the insula have been reported in cases of FM, with higher levels of strength with reference to the connectivity between the right inferior parietal sulcus and the insula cortex (van Ettinger-Veenstra et al., 2020). The functional role of the insula and its connections to sensations relating to the body are well known (Karnath et al., 2005; Moro et al., 2016; Pacella et al., 2019) and seem to concern both its fundamental role in the central processing of interoceptive signals from the body (Craig, 2009) and its contribution to the salience network of the brain (Menon and Uddin, 2010). Indeed, over-focalisation on bodily sensations (Rost et al., 2017) and a perceptual style involving the amplification of interoceptive stimulation (Borg et al., 2018; Martínez et al., 2018) have been reported in FM patients, but these are not associated with greater interoceptive accuracy (Borg et al., 2018).

### Corporeal Illusion and Clinical Variables

No correlations were found between the severity of symptoms or their impact on autonomy (as measured by the SSS, the WPI, and the FIQ) and the various types of corporeal illusions, or between these latter and the intensity of neuromuscular, neuropathic or visceral pain. In the FM group, the only correlations found were between age and feelings of body loss and between age and neuropathic pain.

This was an unexpected result considering that, in the C group, a correlation with the severity of pain was recorded. Furthermore, distortions in body size, weight and localisation have been found to be associated with pain in previous studies (Valenzuela-Moguillansky et al., 2013; Teodoro et al., 2018 for a review). It is possible that the setting or the timing of the interview have an effect and that the link between clinical variables only becomes evident when the patients are interviewed in conjunction with an episode of pain (as for example in Valenzuela-Moguillansky et al., 2013, in which interviews involving the elicitation of pain were used). At the same, it might be worth considering whether the absence of any impact relating to anxiety and depression on corporeal illusions may depend on the specific sample of patients who took part in this study, since none of them were undergoing a painful episode. Anxiety and depressive symptoms are reported as being relatively high among FM patients (Malt et al., 2002; Hadlandsmyth et al., 2019), but no differences were recorded in our sample in comparison to the healthy controls.

Interestingly, somatoparaphrenic-like sensations directly correlate with education in the C group. Relationships between education and bodily illusions have been rarely investigated, probably because the standards of education in experimental studies are usually matched between groups. However, recent studies with the Rubber Hand Illusion are showing that interindividual differences such as education might have an impact on bodily illusions (Haans et al., 2012; Kállai et al., 2015; Burin et al., 2019; Lush et al., 2020, 2021; Romano et al., 2021). However, these interindividual characteristics are not sufficient to explain the individual responses to the induced illusion (David et al., 2013; Scandola et al., 2014; Ehrsson et al., 2021). A possible explanation for this effect is that more educated people show a greater “Openness to Experience” trait of personality, that also involves a leaning toward non-traditional values and experiences (Schretlen et al., 2010). This might lead to the tendency to acceptance of unusual bodily sensations. This is indeed only a speculative hypothesis, as in this study these data on personality traits were not collected

### Motor Imagery

Another result of this study regards the evidence found of specific disorders in motor imagery in FM patients. These involve both the capacity to visually imagine one’s own body while performing an action and the ability to imagine the kinematic sensations produced by that action. In particular, they showed a reduced motor imagery both in the kinaesthetic and third-person perspective imagery. In the FM group, these capacities were reduced in correlation with the loss of functional abilities (FIQ), while no correlations were found with the severity of symptoms and pain intensity, as occurred in the case of the controls. In addition, the FM patient’s history of illness (in terms of the time interval between the appearance of the first symptoms and the degree of depression) correlated with their visual motor imagery disorders.

Motor imagery is closely connected to action execution, as demonstrated by neuroimaging results showing that MI involves neural structures largely overlapping with those involved in actually performing the imagined movements, in particular the pre-motor areas, the left intraparietal sulcus, and subcortical structures such as basal ganglia and cerebellum (Bonda et al., 1995; Decety, 1996; Gerardin et al., 2000; Corradi-Dell’Acqua et al., 2009). The inherent link between motor imagery and action execution has been confirmed in studies showing that MI is altered in a number of pathological conditions characterised by an impairment of the ability to actually perform actions such as locked-in syndrome (Conson et al., 2008), spinal cord injury (Scandola et al., 2017a) amyotrophic lateral sclerosis (Fiori et al., 2013), and chronic pain conditions (Schwoebel et al., 2001; Coslett et al., 2010). Our results suggest that rather than the pain itself, it is the functional deficits which induce motor imagery deficits in FM patients. This would be also consistent with results relating to spinal cord injured patients, which indicate that disorders in motor imagery specifically refer to the actions that have become impossible to execute due to paralysis (Pernigo et al., 2012; Scandola et al., 2017b). The direction of this relationship has yet to be fully understood, however, since although it is possible that functional deficits mediate a reduction in motor imagery, the opposite is also plausible, that is, that motor imagery disorders influence functional abilities.

## Conclusion

The main limitation of the study concerns the number of participants which was not very large in terms of the methodology used which was based on a verbal interview (BoFI-FM) and a self-judgment task (VMIQ). However, the sample seems to be sufficiently representative of the clinical population if one considers the strict inclusion and exclusion criteria for the two groups and the in-depth clinical assessment administered that included functional and emotional measures. In addition, only women were recruited for the study, due to the rarity of the syndrome among males. This did not allow testing for gender differences. Further studies are needed to confirm these preliminary results and compare subjective reports with objective measures of body representations and somatosensory and interoceptive sensitivity. It was not possible to identify the potential presence of a topography relating to corporeal illusions and motor imagery deficits. In other words, it was not established whether the body representation and motor imagery disorders were specific to the painful body parts or were generalised to the whole body. This limitation is inherent to the specific clinical condition, since FM is characterised by widespread pain that involves various different body parts, without a specific topographic organisation—something which is, in contrast, observable, for example, in focal dystonia, or chronic regional pain. It is thus extremely difficult to trace a topography of body representation disorders. Finally, no measure of interoception was collected.

In conclusion, the results indicate that bodily self and motor imagery disorders are two features of FM and that these may be investigated in clinical settings using specific instrumentation. This may, at least in part, explain the higher frequency of falls in these patients which has previously been associated with an altered body schema (Jones et al., 2009; Meireles et al., 2014). From a more general, theoretical perspective, our results demonstrate that FM is a complex, multifaceted syndrome and that body representation disorders, although concurrent, are, however, independent from pain.

## Data Availability Statement

The original contributions presented in the study are publicly available. This data can be found here: osf.io/vxgqj.

## Ethics Statement

The studies involving human participants were reviewed and approved by Comitato etico per la Sperimentazione Clinica (CESC) delle Province di Verona e Rovigo. The patients/participants provided their written informed consent to participate in this study.

## Author Contributions

MS: study ideation and planning, data collection, statistical analyses, and writing. GP: study planning, control recruitment, and data collection. GL: data collection. EP: patient recruitment and clinical assessment. VS: patient recruitment, clinical assessment, and writing. VM: study ideation and planning, data collection, writing, and supervision of all phases of the study. All authors contributed to the article and approved the submitted version.

## Conflict of Interest

The authors declare that the research was conducted in the absence of any commercial or financial relationships that could be construed as a potential conflict of interest.

## Publisher’s Note

All claims expressed in this article are solely those of the authors and do not necessarily represent those of their affiliated organizations, or those of the publisher, the editors and the reviewers. Any product that may be evaluated in this article, or claim that may be made by its manufacturer, is not guaranteed or endorsed by the publisher.
