# Supplementary material for: Bodily Illusions and Motor Imagery in Fibromyalgia
Source: Front Hum Neurosci. 2022 Jan 20;15:798912. doi: 10.3389/fnhum.2021.798912 (PMC8811121; doi:10.3389/fnhum.2021.798912)
Supplement: Supplementary file 1 [file Data_Sheet_1.PDF]

Supplementary Material

Corporal illusions and motor imagery in fibromyalgia

Scandola M[1], Pietroni G[1], Landuzzi G[2], Polati E. 3, Schweiger V[3], Moro V[1*]

A- Clinical features of pain in FM and control group

	FM		C	
musculoskeletal pain	intensity	n.	intensity	n.
Is it sore?	6.15	26	2,00	11
Is it dull?	6.45	24	3.97	19
Is it a cramping pain?	0.57	2	0.57	3
Does it tremble?	1.33	5	0.13	1
Does it vibrate?	1.66	6	0.17	1
Does it throb?	3.1	12	1.6	9
Is it a dull beating pain?	4.17	16	1.77	10
Is it a hammering pain?	3.13	11	1.33	9
visceral pain				
Is it sore?	3.2	13	0.27	1
Is it dull?	3.17	14	0.87	4
Is it a cramping pain?	3.77	14	0.73	4
Does it tremble?	1.03	4	0	0
Does it vibrate?	1.27	6	0.13	1
Does it throb?	3.33	13	0.83	4
Is it a dull beating pain?	3.2	14	0.27	1
Is it a hammering pain?	2.8	12	0.53	2
Is it associated with migraine?	1.93	8	1.37	6
Is it associated with hypertension?	0.87	3	0	0
Is it associated with bradycardia?	0.73	3	0.2	1
Is it associated with sweating?	2.9	12	0.47	3
Is it associated with urinary retention?	1.07	4	0	0
neuropathic pain				
Is it sharp?	2.33	9	0.63	2
Does it burn?	4.27	15	0.3	2
Is it dull?	2.7	11	0.27	2

Is it cold?	0.47	3	0.07	1
When you feel this pain, is your skin hypersensitive to light touching and rubbing?	3.63	15	0.7	3
Is it tingly?	0.93	4	0	0
Is it similar to an electrical shock?	2.77	11	0.93	5

Table SM-A. clinical features of the three typologies of pain (musculosckeletal, visceral and neuropatic pain) as reported by the two groups of participants. Intensity = mean of the intensity as reported by participants for each question.

B- Frequencies of corporal illusions symptoms in Fibromyalgic patients and controls

**Body Feelings and Illusions**		FM	C
		n.30	n.30
1	Have you ever felt strange sensations in your body?	ns	ns
	Body form and integrity		
1.1.	Does it ever feel like any body parts do not belong to you? (DSO)	56.67	10
1.2.	Does it ever feel like your arms are not attached to your shoulders? (DSO)	46.67	3.3
1.3.	Does it ever feel like your legs are not attached to your hips? (SP)	43.3	10
1.4.	Do you ever feel like your legs/arms are elsewhere in the room/in space?	6.67	0
1.5.	Do you ever feel that a part of your body (e.g. your arms or legs) are missing? (BL)	23.33	0
1.6	Do you ever feel that a part of your body (e.g. your arms or legs) are disappeared? (BL)	30	3.33
1.7.	Do you ever feel that your legs have become longer?	0	0
1.8.	Do you ever feel that your arms have become longer?	6.67	0
1.9	Do you ever feel any body parts swelling (IM)	86.67	40
1.10	Do you ever feel like any parts of your body have become smaller?	16.67	0
1.11	Do you ever feel the desire not to have a particular body part? (BL/MP)	63.33	40
1.12	Does it ever feel like some body parts are alien or foreign? (SP)	23.33	0
1.13	Do you ever feel hate for any body parts? (MP)	60	30
	Body and body part positions and illusory movements		
2.1	Does it ever feel like any parts of your body (e.g. arms or legs are in a different position with respect to your real posture? (BPM)	30	10
2.2.	Does it ever feel like you are in a different position with respect to your real posture? (BL)	30	3
2.3.	Does it ever feel like your knees and hips are bent when instead they are totally extended? (BPM)	20	6.67
2.4.	Does it ever feel like your toes are in a strange position, for example curved inwards? (SP)	33	6.67
2.5.	Does it ever feel like any body parts move involuntarily? (IM)	43.33	20
2.6.	Do you ever have the feeling that your muscles are moving with subsequent tiredness? (IM)	26.67	3.33

2.7	Does it ever feel like each digit was twisted so that each toe or finger points in a different direction?	16.67	0
2.8.	Does it ever feel like your fingers or toes are clenched or overlapping one other? (BMP)	23.33	3.33

Table SM-B. The percentage of patients in the two groups who report symptoms for the single items of the questionnaire. See the text for the statistical comparisons of frequencies

C- Correlations between corporal illusions, motor imagery and clinical variables

Correlation table for Fibromyalgia participants

	BL	IM	BPM	AR	DSO	SP	FIQ	WPI	SSS	P-MS	P-Vis	P-Neu	Symp_Int	VMIQ EVI	VMIQ KIN	Anx	Dep	Edu	Age	Sport Past	Sport Now
IM	0.43*																				
BPM	0.15	0.02																			
MP	0.03	0.28	0.23																		
DSO	0.11	0.46*	-0.18	0.48**																	
SP	0.00	0.62***	-0.05	0.49**	0.66***																
FIQ	0.06	0.06	-0.05	-0.19	-0.15	0															
WPI	-0.02	0.03	-0.3	-0.22	0.05	0.06	0.59***														
SSS	0.09	0.19	-0.3	-0.17	0.07	0.13	0.46*	0.3													
P-MS	0.08	-0.19	-0.32	-0.22	-0.02	-0.22	0.41*	0.08	0.12												
P-Vis	-0.02	0.05	0.17	-0.2	-0.12	-0.21	0.2	0.11	0.14	-0.04											
P-Neu	0.13	0.14	-0.03	-0.01	-0.04	0.09	0.35	0.37*	-0.02	0	0.02										
Symp_Int	-0.07	-0.21	0.1	-0.14	0.02	-0.12	0.04	-0.22	-0.24	0.24	-0.09	0.13									
VMIQ EVI	-0.12	0.11	0.02	-0.19	-0.22	0.01	0.38*	0.2	0.12	0.16	0.16	0.1	-0.39*								
VMIQ KIN	-0.16	0.16	-0.11	-0.27	-0.2	0.01	0.42*	0.12	0.19	0.29	0.02	0.06	-0.21	0.77***							
Anx	-0.25	0.18	-0.08	0.27	0.28	0.21	-0.02	0.06	-0.23	0.1	0.2	-0.31	-0.06	0.16	-0.01						
Dep	-0.21	0.24	0.07	0.12	0.35	0.25	0.32	0.17	0.14	0.1	0.33	0.19	-0.11	0.51**	0.42*	0.37*					
Edu	0.19	0.27	0.22	0.05	0.08	0.02	0.07	-0.33	0.32	0	0.22	-0.04	0.25	-0.06	0.02	0.03	0.13				
Age	-0.5**	-0.07	-0.01	-0.04	-0.12	0.19	0.11	0.25	-0.2	-0.13	-0.08	0.38*	0.17	0.3	0.17	0.09	0.2	-0.35			
Sport Past	-0.12	-0.06	0.08	-0.04	-0.14	0.08	0.07	-0.23	-0.01	0.16	-0.19	0.02	0.19	-0.05	0.13	-0.1	-0.03	0.41*	-0.01		
Sport Now	0.01	0.12	-0.04	-0.36	-0.04	-0.15	0.13	-0.09	0.14	0.14	0.35	0.14	0.26	0.09	0.07	-0.03	0.14	0.29	-0.16	0.11	
Job	-0.08	0.08	0.15	0.02	-0.32	-0.2	0.04	-0.23	0.1	0.11	0.17	-0.33	-0.01	0.11	0.23	0.19	-0.11	0.43*	-0.16	0.4*	0.21

Table SM-C1. FM Patients. DSO = disownership-like sensations; SP = somatoparaphrenia-like sensations; BL= body loss; IM = illlusory movements; AR= Aversive Responses; BPM= Body part misperceptions; FIQ = ; WPI = Widespread Pain Index; SSS = Symptom Severity Scale; P-MS = Muscolo-skeletal Pain; P-Vis = Visceral Pain; P-Neu = Neuropathic Pain; Sympt_Int = Interval since the first symptoms appeared (years); VMIQ EVI = Vividness of motor Imagery Questionnaire, External Visual Imagery; VMIQ KIN = Vividness of motor Imagery Questionnaire, Kinaesthetic Imagery; Anx = HADS Anxiety subscale; Dep = HADS Depression subscale; Edu = education (years); Sport Past = sport previous the occurrence of fibromyalgia (1 = yes, 0 = no); Sport Now (1 = yes, 0 = no); Job (1 = yes, 0 = no); *** = p < 0.001; ** = p < 0.01; * = p < 0.05.

Correlation table for Control participants

	BL	IM	BPM	AR	DSO	SP	FIQ	WPI	SSS	P-MS	P-Vis	P-Neu	VMIQ EVI	VMIQ KIN	Anx	Dep	Edu	Age	Sport Now
**IM**	-0.33																		
**BPM**	0.11	-0.09																	
**MP**	0.04	0.27	-0.36																
**DSO**	-0.06	0.17	0.27	-0.27															
**SP**	0.14	0.08	-0.11	-0.04	0.57***														
**FIQ**	-0.21	0.25	0.00	0.37*	0.13	0.28													
**WPI**	-0.15	0.3	0.07	0.42*	0.31	0.28	0.57**												
**SSS**	-0.39*	0.39*	-0.31	0.49**	0.07	0.12	0.62***	0.44*											
**P-MS**	-0.14	0.30	0.17	0.2	0.29	0.14	0.4*	0.52**	0.36*										
**P-Vis**	-0.10	-0.32	-0.04	0.00	-0.07	-0.21	-0.01	-0.06	0.04	0.01									
**P-Neu**	0.37*	0.01	0.02	0.19	-0.17	-0.03	0.05	0.16	0.00	0	0.08								
**VMIQ EVI**	-0.30	0.11	-0.16	0.28	0.00	-0.05	0.3	0.36*	0.67***	0.01	0.03	-0.13							
**VMIQ KIN**	-0.14	0.02	0	0.26	-0.05	-0.17	0.44*	0.35	0.52**	0.31	-0.06	0.00	0.67***						
**Anx**	-0.15	0.31	-0.32	0.33	-0.06	0.04	0.04	0.37*	0.33	0.2	0.04	-0.01	0.19	-0.13					
**Dep**	0.02	0.24	-0.18	-0.04	-0.14	-0.17	0.06	0.00	0.2	0.01	-0.04	0.25	0.21	0.3	0.13				
**Edu**	0.02	-0.18	-0.13	0.17	-0.37*	-0.49**	-0.37*	-0.14	-0.1	-0.1	0.05	-0.1	0.08	0.04	0.03	-0.02			
**Age**	0.00	0.02	-0.01	0.00	0.09	0.05	0.11	-0.06	-0.29	0.1	0.17	-0.01	-0.33	0.1	-0.35	0.09	-0.09		
**Sport Now**	0.00	-0.23	-0.06	0.12	-0.06	0.00	-0.26	0.05	-0.13	-0.02	0.36*	0.24	-0.02	-0.07	0.02	-0.25	-0.02	0.32	
**Job**	-0.14	-0.22	0.12	-0.02	-0.11	-0.29	-0.34	-0.39*	-0.21	-0.27	0.22	-0.28	-0.14	-0.31	0.04	-0.36	0.47**	-0.23	-0.12

Table SM-C2. DSO = disownership-like sensations; SP = somatoparaphrenia-like sensations; BL= body loss; IM = illlusory movements; AR= Aversive Responses; BPM= Body part misperceptions; FIQ = ; WPI = Widespread Pain Index; SSS = Symptom Severity Scale; P-MS

= Muscolo-skeletal Pain; P-Vis = Visceral Pain; P-Neu = Neuropathic Pain; Sympt_Int = Interval since the first symptoms appeared (years); VMIQ EVI = Vividness of motor Imagery Questionnaire, External Visual Imagery; VMIQ KIN = Vividness of motor Imagery Questionnaire, Kinaesthetic Imagery; Anx = HADS Anxiety subscale; Dep = HADS Depression subscale; Edu = education (years); Sport Past = sport previous the occurrence of fibromyalgia (1 = yes, 0 = no); Sport Now (1 = yes, 0 = no); Job (1 = yes, 0 = no); *** = $p < 0.001$; ** = $p < 0.01$; * = $p < 0.05$.
